# Unusual domain architecture of aminoacyl tRNA synthetases and their paralogs from *Leishmania major*

**DOI:** 10.1186/1471-2164-13-621

**Published:** 2012-11-14

**Authors:** V S Gowri, Indira Ghosh, Amit Sharma, Rentala Madhubala

**Affiliations:** 1 School of Life Sciences, Jawaharlal Nehru University, New Delhi, 110 067, India; 2 School of Computational and Integrative Sciences, Jawaharlal Nehru University, New Delhi, 110 067, India; 3 Structural and Computational biology, International Centre for Genetic Engineering and Biotechnology, New Delhi, 110 067, India

**Keywords:** Aminoacyl tRNA synthetases, Paralog, Editing domains

## Abstract

**Background:**

*Leishmania major*, a protozoan parasite, is the causative agent of cutaneous leishmaniasis. Due to the development of resistance against the currently available anti-leishmanial drugs, there is a growing need for specific inhibitors and novel drug targets. In this regards, aminoacyl tRNA synthetases, the linchpins of protein synthesis, have received recent attention among the kinetoplastid research community. This is the first comprehensive survey of the aminoacyl tRNA synthetases, their paralogs and other associated proteins from *L. major*.

**Results:**

A total of 26 aminoacyl tRNA synthetases were identified using various computational and bioinformatics tools. Phylogenetic analysis and domain architectures of the *L. major* aminoacyl tRNA synthetases suggest a probable archaeal/eukaryotic origin. Presence of additional domains or N- or C-terminal extensions in 11 aminoacyl tRNA synthetases from *L. major* suggests possibilities such as additional tRNA binding or oligomerization or editing activity. Five freestanding editing domains were identified in *L. major*. Domain assignment revealed a novel asparagine tRNA synthetase paralog, asparagine synthetase A which has been so far reported from prokaryotes and archaea.

**Conclusions:**

A comprehensive bioinformatic analysis revealed 26 aminoacyl tRNA synthetases and five freestanding editing domains in *L. major*. Identification of two EMAP (endothelial monocyte-activating polypeptide) II-like proteins similar to human EMAP II-like proteins suggests their participation in multisynthetase complex formation. While the phylogeny of tRNA synthetases suggests a probable archaeal/eukaryotic origin, phylogeny of asparagine synthetase A strongly suggests a bacterial origin. The unique features identified in this work provide rationale for designing inhibitors against parasite aminoacyl tRNA synthetases and their paralogs.

## Background

Aminoacyl tRNA synthetases (aaRSs) are indispensable components of protein synthesis. They covalently append amino acids to their cognate tRNA. Most organisms possess separate tRNA synthetases for each of the 20 standard amino acids. There are two classes of aminoacyl tRNA synthetases each comprising of ~10 tRNA synthetase enzymes - Class I tRNA synthetases contain the classical Rossmann-nucleotide binding catalytic domain with two highly conserved 'HIGH' and 'KMSKS' catalytic motifs which are critical for their function [1]. Class II enzymes contain a central antiparallel β-sheet flanked by α-helices. Despite these structural and sequence differences, both tRNA synthetases catalyse the same two step reaction. The first step involves activation of the amino acid by ATP to form aminoacyl adenylate. The second step is the attachment of the aminoacyl group to the cognate tRNA. While Class I attaches aminoacyl group to the 2'-hydroxyl group of tRNA, Class II synthetases attach them to 3'-hydroxylgroup of tRNA. The 3-D structure and the specific anticodon in the tRNA determine the specificity of tRNA synthetases. Most eukaryotes carry two genes for each of the 20 standard aminoacyl tRNA synthetases (cytosolic and mitochondrial). However, trypanosomatids carry only a single copy per aminoacid except for Asp, Trp and Lys [2,3].

Gene knock out studies of the Trypanosomal histidyl tRNA synthetase showed a complete arrest of growth in the bloodstream forms of the parasite suggesting an essential role in cell survival [4]. Mammalian methionyl tRNA synthetase provides a cytosolic anchoring site for Aminoacyl tRNA synthetase Interaction Multifunctional Protein-3 (AIMP-3/p18), a potent tumor suppressor in addition to its essential role in initiating translation [5]. Participation of aminoacyl tRNA synthetases in cell apoptosis, rRNA synthesis, RNA trafficking, multisynthetase enzyme complex formation supplement their essential role in protein synthesis [6,7]. As an inevitable component of protein synthesis, aminoacyl tRNA synthetases have been important antibacterial drug targets. An important example of aaRS inhibitor is provided by the antibiotic mupirocin which selectively inactivates bacterial Isoleucyl-tRNA synthetase (IleRS) [8,9]. Four distinct aminoacyl tRNA synthetases in *Anabaena sp.* PCC 7120 contain a novel CAAD domain that bears putative transmembrane helices. Domain deletion studies indicate its essential role in membrane anchoring and a purely structural role and do not alter the catalytic properties of the enzyme [10]. Eukaryotic tRNA synthetases, unlike their prokaryotic counterparts carry additional domains or extensions in their N- or C-terminal regions which mediate protein-protein interaction or involved in tRNA binding. A comprehensive computational analysis of the aminoacyl tRNA synthetases of *Plasmodium falciparum* reveals novel domain architectures. Phylogenetic analyses of several Pf aaRSs reconcile their evolutionary link to plants and bacteria [11]. Recently, the expression and localization profiles of the cis- and trans- aaRS editing domains of *P. falciparum* showed an uneven distribution of 8 aaRS editing domains in the different cellular compartments [12].

Leishmaniasis is one of the deadly diseases caused by the different species of *Leishmania*. Increasing resistance to presently available anti-leishmanial drugs poses a need for identification of novel drug targets as well as specific inhibitors for treating leishmaniasis. In this regard, aminoacyl tRNA synthetases, the versatile players of protein translation machinery have received attention in kinetoplastid research community [13]. Very recently, crystal structure of a methionyl-tRNA synthetase [14] and a novel pseudodimeric structure of a tyrosyl tRNA synthetase from *Leishmania major* have been solved [15]. Substantial differences between the human tRNA synthetases and the *L. major* tRNA synthetase homologue promise a rationale for designing inhibitors to selectively target the parasite enzyme. A comprehensive bioinformatic analysis employing the profile-based hidden markov model (HMM) has identified aaRSs and aaRS related proteins from *L. major.* The sequence features and novel domain architectures of aaRSs from *L. major* were analyzed using a combination of BLAST and HMM search tools. Domain assignment revealed a novel asparagine tRNA synthetase (AsnRS) paralog Asparagine synthetase A (AsnA) which has been so far reported from prokaryotes and archaea and has been shown to be absent in eukaryotes. We for the first time report the phylogeny and structural analysis of a eukaryotic AsnA from *L. major*.

## Results and discussions

A total of 26 aminoacyl tRNA synthetases (11 Class I; 14 Class II; 1 non-standard) were identified in *L. major* (Table 1) using Hidden Markov Models (HMMs). Like other trypanosomatids [2,3], *L. major* also has a single copy of the tRNA synthetases except for Asp, Lys, Trp as well as Pro. The presence of the synthetase and anticodon binding domains were confirmed using the Conserved Domains Database (CDD) domain assignments from NCBI. Based on the generic domain architecture, 25 *L. major* sequences identified using the HMM searches could be certified as authentic aaRSs (Table 1). Among the tRNA synthetase related proteins, LmjF.16.1130 and LmjF.22.0470 contain only an RNA binding domain/Myf domain. However, BLAST sequence search against PDB database identified human EMAP II-like sequences (E-value: 2e-21; 37%) as the top hit suggesting their sequence relationship with the EMAP II-like sequences such as P43 from human, Arc1p from yeast, Trbp111 from *A. aeolicus* etc. Both LmjF.16.1130 and LmjF.22.0470 also contain a modified heptapeptide motif that has been shown to be essential for the cytokine activity in the human EMAP II-like protein [16]. The presence of ‘ELR’ motif at the N-terminus has also been shown to be potent promoters of angiogenesis [16]. Aminoacyl tRNA synthetase sequences of Cys (LmjF.12.0250), Asn (LmjF.34.2340), Lys (LmjF.15.0230) and Tyr (LmjF.14.1370) [15] possess an “ELR” motif at the N-terminus. LmjF.26.0830 contains only the Class II synthetase catalytic core with all the three active site motifs conserved. BLAST search against PDB database identified the *E. coli* Asparagine synthetase A structure as the single hit with a reliable statistical value (E-value: 8e-111). LmjF.26.0830 shares 58% sequence identity with the *E. coli* Asparagine synthetase A.

**Table 1:** List of all the aminoacyl tRNA synthetases and their associated proteins, aaRS paralogs and editing domains with their CDD domain assignments and subcellular localization

	**Gene name**	**Length (aa)**	**HMM based function assignment**	**E-value#**	**Swissprot function assignment**	**Subcellular location (PSORT II)**	**CDD based domain assignment**
CLASS I aaRS	LmjF.12.0250	784	Cysteinyl-tRNA synthetase	1.00E-191	Cysteinyl-tRNA synthetase	Cytosol	CysRS_Core; anticodon_la_like_superfamily
	LmjF.15.1440	570	Glutaminyl-tRNA synthetase	8.70E-208	Glutaminyl-tRNA synthetase	Cytosol	GlnRS_core; GlnRS_core; tRNA_synt_Ic_C_superfamily
	LmjF.30.3240	594	Glutaminyl-tRNA synthetase	7.00E-079	Glutaminyl-tRNA synthetase	Cytosol	GlnRS_core; GlnRS_core; tRNA_synt_Ic_C_superfamily
	LmjF.27.1310	692	Arginyl-tRNA synthetase	2.00E-151	Arginyl-tRNA synthetase	Cytosol	Arg_tRNA_synt_N; ArgRS_Core; anticodon_Ia_like_superfamily
	LmjF.36.5620	1009	Isoleucyl-tRNA synthetase	0	Isoleucyl-tRNA synthetase	Cytosol	IleRS_Core; Anticodon_Ia_like_superfamily
	LmjF.13.1100	1075	Leucyl-tRNA synthetase	3.70E-140	Leucyl-tRNA synthetase	Cytosol	IleRS_Core; Anticodon_Ia_like_superfamily
	LmjF.21.0810*	747	Methionyl-tRNA synthetase	6.00E-145	Methionyl-tRNA synthetase	Cytosol	MetRS_Core_superfamily; Anticodon_Ia_Met
	LmjF.23.0300	412	Tryptophanyl-tRNA synthetase	1.90E-062	Tryptophanyl-tRNA synthetase	Cytosol	TrpRS_core
	LmjF.29.0060	480	Tryptophanyl-tRNA synthetase	1.70E-095	Tryptophanyl-tRNA synthetase	Mitochondrial	TrpRS_core
	LmjF.14.1370*	682	Tyrosyl-tRNA synthetase	1.10E-051	Tyrosyl-tRNA synthetase	Endoplasmic reticulum	TyrRS_core; TyrRS_core
	LmjF.30.3130	967	Valyl-tRNA synthetase	0	Valyl-tRNA synthetase	Cytosol	ValRS_core; anticodon_Ia_Val; Val_tRNA_synt_C_superfamily
CLASS II aaRS	LmjF.22.1540	962	Alanyl-tRNA synthetase	3.10E-262	Alanyl-tRNA synthetase	Cytosol	AlaRS_core; tRNA_SAD
	LmjF.30.0460	550	Aspartyl-tRNA synthetase	3.90E-106	Aspartyl-tRNA synthetase	Cytosol	AspRS_cyto_N; AsxRS_Core
	LmjF.21.0895	641	Aspartyl-tRNA synthetase	5.70E-097	Aspartyl-tRNA synthetase	Mitochondrial	AspRS_cyto_N; ClassII_aaRS_like_core_superfamily; ClassII_aaRS_like_core_superfamily
	LmjF.34.2340	890	Asparaginyl-tRNA synthetase	2.40E-144	Asparaginyl-tRNA synthetase	Cytosol	tRNA_synt_Ic_R1_superfamily; AsnRS_cyto_like_N; AsxRS_core
	LmjF.36.3840	628	Glycyl-tRNA synthetase	5.10E-056	Glycyl-tRNA synthetase	Cytosol	GlyRS_core; HGTP_anticodon
	LmjF.30.0130	586	Lysyl-tRNA synthetase	8.20E-184	Lysyl-tRNA synthetase	Cytosol	LysRS_N; LysRS_Core
	LmjF.15.0230	536	Lysyl-tRNA synthetase	9.30E-148	Lysyl-tRNA synthetase	Cytosol	LysRS_N; LysRS_Core
	LmjF.19.1040	633	Phenylalanyl-tRNA synthetase	3.20E-053	Phenylalanyl-tRNA synthetase	Cytosol	B3_4_superfamily; B5_superfamily; PheRS_beta_core
	LmjF.32.0870	499	Phenylalanyl-tRNA synthetase	5.90E-052	Phenylalanyl-tRNA synthetase	Cytosol	PheRS_alpha_core
	LmjF.18.1210	731	Prolyl-tRNA synthetase	1.30E-188	Prolyl-tRNA synthetase	Cytosol	Ybak_like_superfamily; ProRS_arch_euk; HGTP_anticodon_superfamily
	LmjF.18.1220	731	Prolyl-tRNA synthetase	1.20E-190	Prolyl-tRNA synthetase	Cytosol	Ybak_like_superfamily; ProRS_arch_euk; HGTP_anticodon_superfamily
	LmjF.11.0100	474	Seryl-tRNA synthetase	4.40E-135	Seryl-tRNA synthetase	Cytosol	Seryl_tRNA_N_superfamily; SerRS_Core
	LmjF.30.0630	473	Histidyl-tRNA synthetase	2.30E-103	Histidyl-tRNA synthetase	Cytosol	HisRS_Core; HisRS_anticodon
	LmjF.35.1410	787	Threonyl-tRNA synthetase	3.20E-252	Threonyl-tRNA synthetase	Cytosol	TGS_superfamily; tRNA_SAD; ThrRS_Core; HGTP_anticodon
Non-canonical	LmjF.09.0950	595	O-Phosphoseryl-tRNA Selenium transferase	9.10E-127	SLA/LP autoantigen-like protein	Nuclear	Selenium_SpcS
aaRS associated proteins	LmjF.22.0470	426	EMAP-II like	1.20E-023	Hypothetical, Conserved	Extracellular	tRNA_binding_domain_superfamily
	LmjF.16.1130	180	EMAP-II like	1.20E-015	Tyrosyl/methionyl-tRNA synthetase	Cytosol	tRNAbindingdomain_superfamily
AsnRS paralog	LmjF.26.0830	353	Asparagine Synthetase A	2.00E-002	Aspartate, ammonia ligase	Mitochondrial	ClassII_aaRS_like_core_superfamily
*Trans* Editing domains	LmjF.15.0690	491	AlaX	3.90E-028	Hypothetical, conserved	Cytosol	tRNA_SAD_superfamily
	LmjF.03.0710	253	YbaK	2.20E-002	Hypothetical, conserved	Cytosol	Ybak_like_superfamily
	LmjF.21.0910	275	YbaK	7.20E-003	Hypothetical, conserved	Cytosol	Ybak_like_superfamily
	LmjF.36.2730	152	Dtda	1.00E-026	D-tyrosine deacylase	Cytosol	Dtyr_deacylase
	LmjF.34.3360*	211	Dtda	5.40E-020	Hypothetical, conserved	Cytosol	Dtyr_deacylase_superfamily

^*^ indicates *L. major* sequences whose crystal structures are available ^#^ E-value (Expectation value) is an indication of the significance of a hit to the HMM Model queried. This gives a more quantitative measure of statistical significance. The lower the E-value, the better is the significance of the hit to the query HMM.

As the key players in protein translation, most organisms require 20 standard aminoacyl tRNA synthetases for protein synthesis. However, indirect routes of GlntRNA^Gln^ and AsntRNA^Asn^ synthesis also exist in many organisms which either completely lack the respective tRNA synthetases or lack them in some specific organelles such as mitochondria [17]. Kinetplastid (*Trypanosoma and Leishmania*) Seryl tRNA synthetases (SerRS) show a close functional and evolutionary relationship to the metazoan SerRS which is supported by the presence of a metazoan-trypanosomatid specific sequence insertion in SerRS [18]. The kinetoplastid SerRS also show high affinity for tRNA^Sec^[18]. Proteins containing non-standard aminoacids such as Sec (Selenocysteine) have been reported from trypanosomatids [19]. The presence of Selenocysteine incorporation is further supported by the presence of a selenophosphosynthetase (LmjF.36.5410), the first enzyme in the selenocysteine tRNA synthesis as well as a selenocysteine specific elongation factor (LmjF.34.2840). LmjF.09.0950, a o-phosphoseryl tRNA(sec) selenium transferase, the third enzyme in the SectRNA^Sec^ synthesis with SepSecS-like domains was also identified using the Hidden Markov Model searches.

**Figure 1:**
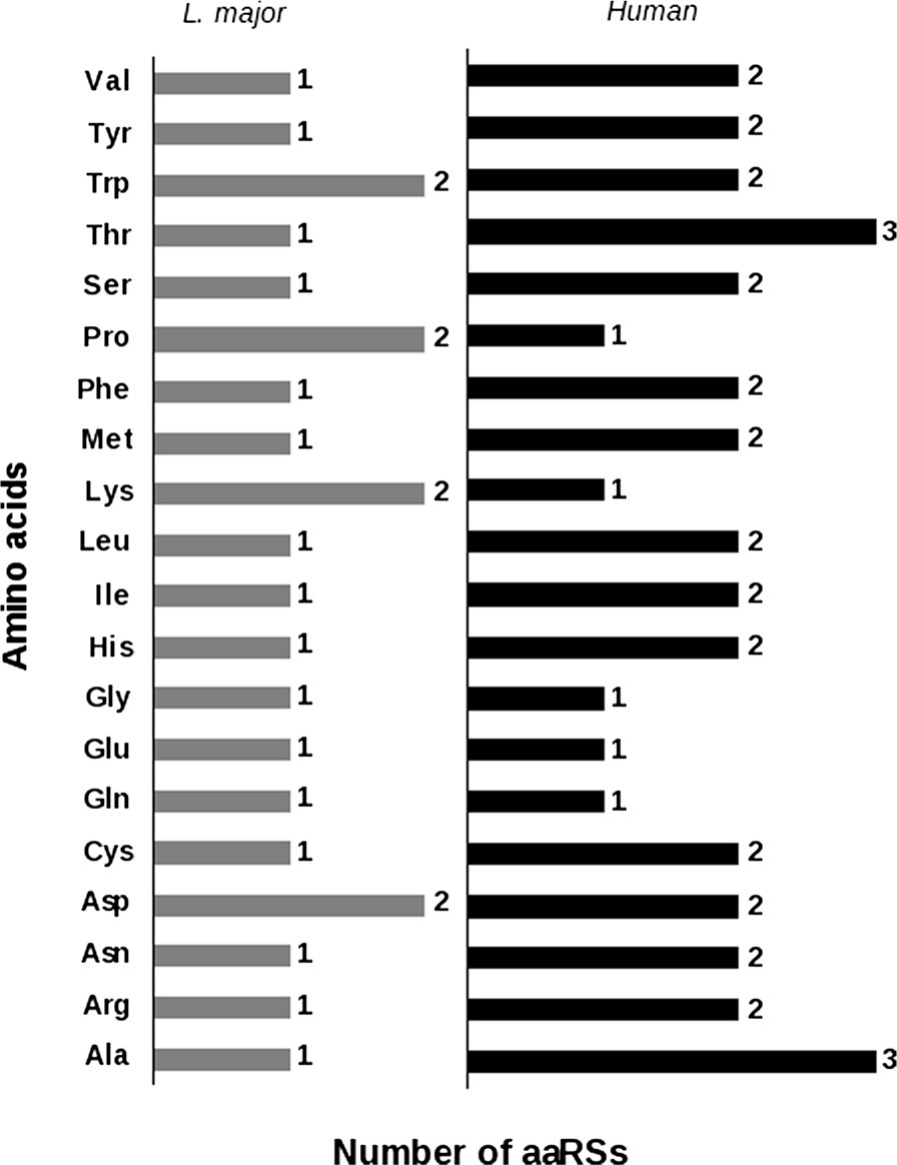
**A panel bar diagram showing the number of the standard aaRSs in *L. major *and human.**

Comparison of the number of aminoacyl tRNA synthetases (20 standard aminoacids) of the human with *L. major* (Figure 1) shows a disparity in the number of aaRS for all the aminoacids except for Gly, Glu and Gln where a single copy is present in both human and *Leishmania*. Two copies one each for cytoplasm and mitochondria of AspRS and TrpRS are present in both human and *L. major.* While human possess a single copy of LysRS and ProRS, *L. major* has two copies of these predicted to be in the cytoplasm. One of the LysRS (LmjF.15.0230) has an “ELR” motif at the N-terminus. The two copies of ProRS from *L. major* are identical copies probably a product of gene duplication. Humans possess the maximum number of alanyl and threonyl tRNA synthetases (3 copies each) compared to *L. major* which has a single copy of each of them. Non-canonical roles of tRNA synthetases require their presence in diverse cellular compartments. Hence, prediction of subcellular localization of the *Lm*aaRSs was done using PSORT-II. 80% of the tRNA synthetases are cytosolic and 10% of them are present in mitochondria according to PSORT II predictions (Table 1). Nuclear localization was predicted for the non-standard o-phosphoseryl tRNA(sec) selenium transferase (LmjF.09.0950). Mitochondrial localization was predicted for 3 proteins corresponding to an AspRS, AsnA (AsnRS paralog) and TrpRS (Table 1). Many proteins that are imported into mitochondrion have targeting signals typically at the N-terminus [20] or C-terminus or protein internal. However, numerous mitochondrial proteins have been shown to be lacking these signals including those proteins that have been shown to be imported into mitochondria in trypanosomes. Examples include the glutamyl and glutaminyl tRNA synthetases from *L. tarentolae*[21] and *T. brucei*[22]. Although, the glutaminyl tRNA synthetases are shown to be absent in their mitochondria, the glutaminyl tRNA synthetase activity has been shown experimentally in both these organisms. Hence, it is possible that the single copy tRNA synthetases are probably transported to mitochondria during translation although PSORT II is unable to predict the possibility of mitochondrial localization for the single copy ones. Trans splicing of a leader sequence to the 5’ end of the mRNA is a common phenomenon among human and protozoa. This results in alternative splicing in these organisms resulting in proteins with different properties such as gain or loss of targeting signals. Such a mapping of the 5’ splice sites using the splice leader trapping method in *T. brucei* resulted in the discovery of nearly 2500 alternative splice events in a stage-regulated manner [23]. The splice sites data for *L. major* at the tritrypdb server suggests an alternate start site as a result of trans splicing in the promastigote stages for several tRNA synthetases including the single copy tRNA synthetases such as the valyl, isoleucyl, leucyl, glutamyl tRNA synthetases [Additional file 1: Table S1].

The tRNA synthetases and other associated proteins such as the EMAP II and the editing domains although present in all the trypanosomatids, show interesting differences between the *Leishmania* and *Trypanosoma* [Additional file 2: Table S2]. There are two AspRS in all the *Leishmania Spp, T. brucei brucei and T. brucei gambiense. However, T. congolense and T. vivax* have only a single copy of AspRS. Furthermore, all the *Leishmania* spp and *Trypanosoma spp* carry a single HisRS except *T. congolense. T. cruzi Non-Esmeraldo strain* and *T. congolense* carry three TrpRS whereas all other trypanostomatids carry two TrpRS. *T. cruzi Non-Esmeraldo strain* lacks a MetRS and GlnRS. Moreover, *T. cruzi Non-Esmeraldo strain* contains only an alpha chain of PheRS and *T. cruzi Esmeraldo strain* lacks an AsnRS and ArgRS. Although several tRNA synthetases are syntenic and conserved in *Leishmania*, the protein expression is regulated at different stages in different *Leishmania* species. For example, SerRS (LmjF.11.0100), LysRS (LmjF.15.230), glutamyl (LmjF.30.3240), AsnRS (LmjF.34.2340), GlyRS (LmjF.36.3840), IleRS (LmjF.36.5620) are regulated predominantly in the promastigotes in *L. major*[24].

### Domain architecture of tRNA synthetases from *L. major*

**Figure 2:**
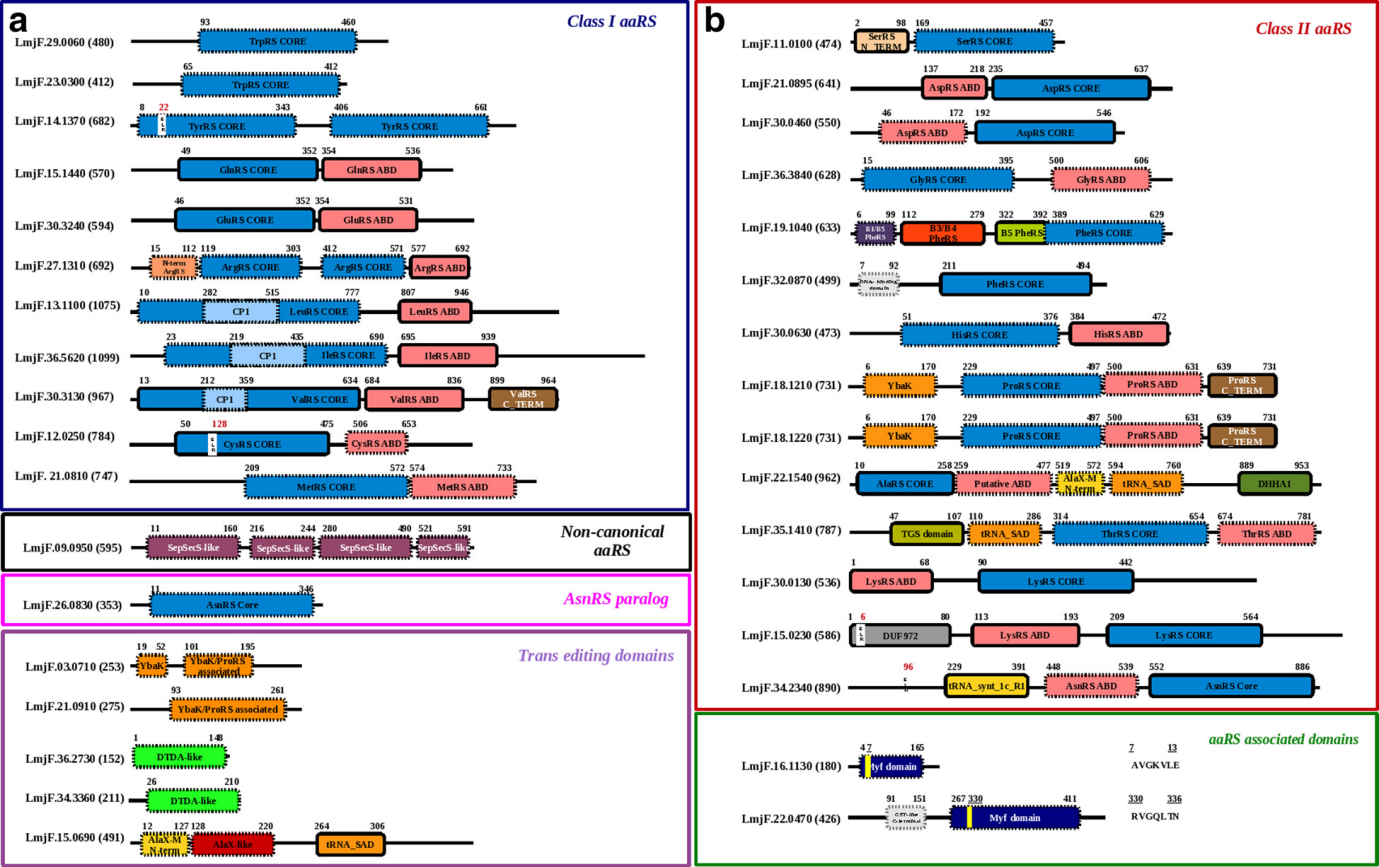
**Domain architecture of (a) Class I and (b) Class II *Lm*aaRSs, aaRS associated proteins, and *trans *editing domains.** The box line type for domain boundaries correspond to the domain prediction procedure (solid line for PFAM HMM predictions; dotted line for SUPERFAMILY based HMM predictions). The beginning of ELR motif is denoted by the residue number in red color font. The beginning of heptapeptide motif is denoted by an underlined residue number. The grey boxes corresponds to lower prediction accuracy (E:value: greater than 1E-05).

All the standard aminoacyl tRNA synthetases contain the synthetase core domain as well as the anticodon binding domain in some order. Hence, the presence of these generic domains helps in distinguishing the aminoacyl tRNA synthetases from the aminoacyl tRNA synthetase associated proteins (aaRS associated proteins) which refers to EMAP II-like proteins containing only the RNA binding domains. In addition to these genericdomains, some of the aminoacyl tRNA synthetases also possess additional domains or extensions tethered to either N- or C-terminus which might be involved in RNA binding or oligomerization. Presence of editing domains ensures the fidelity of protein translation in some tRNA synthetases by hydrolysing the tRNA aminoacylated with non-cognate amino acid [25]. Thus, in addition to the aaRS, the editing domains (both cis- and trans-) are also novel drug targets. The domain architecture of all the aaRSs (including the non-standard o-phosphoseryl tRNA(sec) selenium transferase) with all the additional domains or motifs, *trans* editing domains, aaRS paralogs and other aaRS associated proteins of *L. major* is shown in Figure 2a and b.

Alanyl and threonyl tRNA synthetases often possess a secondary associated domain (tRNA_SAD) containing a HxxxH motif which is typical of a metal dependent hydrolases [26]. Alanyl (LmjF.22.1540) and threonyl tRNA synthetases (LmjF.35.1410) of *L. major* contain this domain. The presence of a tRNA_SAD domain with a conserved HxxxH motif suggests a functionally important hydrolytic activity (Figure 2b). *Lm*ThrRS also contains a TGS domain tethered N-terminus to the tRNA_SAD (Figure 2b). Based on its occurrence in other regulatory proteins, this domain is proposed to bind ligands (most likely nucleotides) [27]. Hence, the TGS domain in *Lm*ThrRS probably has a regulatory role. In addition to an editing domain tRNA_SAD, *Lm*AlaRS has a C-terminal extension (DHHA1 domain). Crystal structure and functional analysis of this C–Ala extension in *A. aeolicus* AlaRS shows that it promotes cooperative binding of the aminoacylation and editing domain to tRNA^Ala^[28].

While the C-terminal extension of *Lm*AlaRS might be involved in oligomerization, N- or C-terminal extensions of SerRS, LeuRS and LysRS have been shown to provide additional tRNA binding to these synthetases [29-31]. The N-terminal extension of *Lm*LeuRS (LmjF.13.1100) is present as insertion in the editing domain denoted as CP1 (Connective Polypeptide) (Figure 2a). Only three LeuRS editing domains have been structurally characterized till date [31,32]. The CP1 of *E. coli* LeuRS lacks this insertion and hence lacks the editing activity as an isolated CP1 domain [33]. Recent crystallographic and biochemical evidences reconcile this observation [31]. *Lm*LeuRS has an N-terminal extension of approximately 35 residues long. Secondary structure prediction of this insertion using PSIPRED server [29] suggests that this N-terminal extension has a helix of ~15 residues long (Additional file 3: Figure S1). Further, sequence comparison of the editing domain of *Lm*leuRS with that of human, *E. coli, A. aeolicus* and *G. lamblia* suggest the T-rich region, GTG motif and the conserved Asp essential for function are all conserved. Sequence-based phylogeny suggests a close evolutionary relationship of *Lm*LeuRSCP1 to *Gl*LeuRSCP1 which has been verified to possess fully functional editing domain in isolation [31]. The antifungal drug (AN2690) binding residues of *C. albicans* LeuRS are also highly conserved in *Lm*LeuRS editing domain. Although *Lm*LeuRS is ~1100 residues long, the presence of a probable functional editing domain in isolation proves it to be a novel drug target and encourages experimental verification for its drug binding abilities.

*L. major* encodes two cytosolic LysRS (LmjF.15.0230 and LmjF.30.0130) (Table 1). One of the *Lm*LysRS (LmjF.15.0230) has an N-terminal extension (DUF972) similar to the mammalian LysRS (Figure 2b). The N-terminal extension of mammalian LysRS has been shown to participate in non-specific tRNA binding [26]. Deletion of this N-terminal extension has been shown to reduce the tRNA binding affinity by 100-fold and hence decreases the aminoacylation of tRNA^lys^ by 3-fold in mammals [29]. Based on the sequence homology of the *Lm*LysRS (LmjF.15.0230) to the mammalian LysRS, the N-terminal extension in LmjF.15.0230 can be expected to participate in a non-specific tRNA binding and could probably play a role in amino acylation activity of this LysRS. LmjF.15.0230 also contains an ‘ELR’ motif at the N-terminal extension. However, chemokine activity of this ‘ELR’ motif in *Lm*LysRS requires experimental verification.

### Stand-alone deacylase/*trans* editing domains in *L. major*

While the pairing of the correct amino acid to their cognate tRNA is done by the aminoacyl tRNA synthetases, faithful translation in protein synthesis is ensured by the presence of editing domains(ED) either tethered to the aaRS (*cis* editing domains) or as free standing editing domains (*trans* editing domains). There are 8 *cis* editing domains tethered to AlaRS, ThrRS, PheRS, LeuRS, IleRS, ValRS, ProRS (both the copies) and 5 *trans* editing domains which includes the AlaX (AlaRS ED), two YbaK-like (ProRS ED) and two D-tyrosyl deacylases (DTDAs) in *L. major.*

The Second Associated domain (tRNA_SAD) of AlaRS/ThrRS are generally tethered to the synthetase core. In *L. major,* in addition to the tethered editing domains in the AlaRS (LmjF.22.1540) and ThrRS (LmjF.35.1410), a freestanding tRNA_SAD (LmjF.15.0690) domain (Figure 2a) was also found. There are two types of standalone AlaX domains: AlaX-M, AlaX-S. Both the domains differ in their metal coordination types (Zn coordination). In AlaX-M, in addition to coordination with the Cysteine residues, there is coordination with a water molecule. However, in AlaX-S, the metal ion is coordinated only by cysteines [34,35]. The standalone domain has all the four cysteines conserved. Sequence based phylogeny suggests that *L. major* tRNA_SAD standalone domain is closer to AlaX-M family (Additional file 4: Figure S2).

In addition to the tethered Ybak/ProX deacylase domains in the two *Lm*ProRS copies, two freestanding YbaK domains (LmjF.03.0710 and LmjF.21.0910) are also found (Figure 2a). Sequence based phylogeny of both tethered and standalone deacylase domains in *L. major* with the available crystal structures of YbaK/ProX domains suggest that the tethered deacylase domains are closer in terms of their amino acid sequence to ProX type which specifically deacylate the misacylated tRNA^Pro^ with Alanine [36,37] while the trans deacylase domains are closer to YbaK-like which deacylate the misacylated tRNA^Pro^ with Cysteine [37,38] (Additional file 5: Figure S3). The *trans* editing domain LmjF.03.0710 lacks the active site lysine which is shown to be critical in *H. influenza* YbaK domain by mutagenesis (K46 in PDB: 1DBX) [38-40]. Thus, only one of the *trans* editing domain of *Lm*ProRS (LmjF.21.0910) which has the active site lysine conserved might be functional.

Detection of D-amino acids in the form of free aminoacids, peptides and proteins in various living organisms from bacteria to human challenges the current concept of protein synthesis [41,42]. D-Tyr tRNA^Tyr^ deacylases (DTDA), a new class of tRNA dependent hydrolases provide a novel checkpoint by recycling the misaminoacylated D-Tyr tRNA^Tyr^. There are two DTDAs in *L. major*. One of the *Lm*DTDA2 (LmjF.34.3360) has a crystal structure solved using the Structural Genomics of Pathogenic Protozoa Consortium (SGPP) (PDB: 1TC5). This sequence is closer to the human, mouse DTDA2 homologue. The other *Lm*DTDA1 homologue (LmjF.36.2730) is closer to the *Pf*DTDA homologue (Additional file 6: Figure S4) whose structure has been solved recently (3KO5). Like other deacylases/editing domains of aaRSs, DTDA has been shown to be a novel drug target in *P. falciparum*[43]. While structural comparison of the *Lm*DTDA2 and *Pf*DTDA1 shows major differences in the length of a specific loop, 3-D structural modeling of *Lm*DTDA1 homologue (LmjF.36.2730) based on the *Pf*DTD template structure and a comparative structural analysis of *Lm*DTDA1 with a perspective of inhibitor design is commendable.

### Novel aminoacyl tRNA synthetases from *L. major*

**Figure 3:**
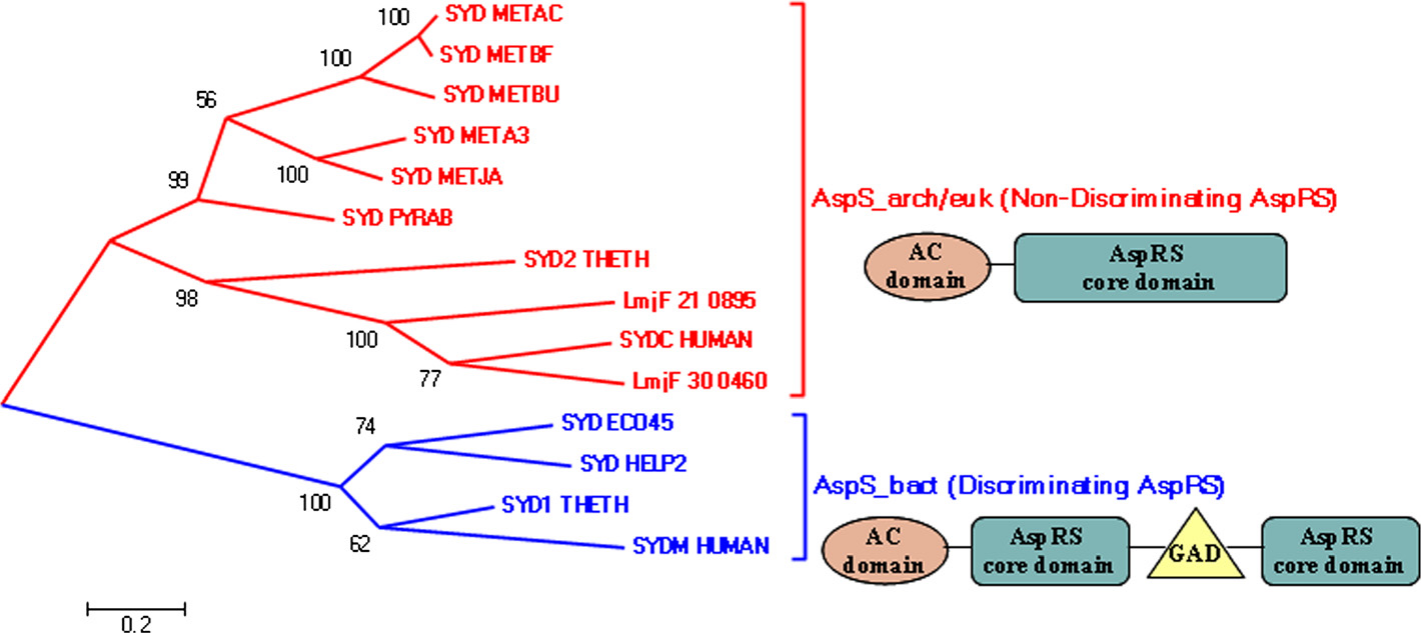
**Sequence based phylogeny of aspartyl tRNA synthetases constructed using MEGA v5.0 using Maximum Likelihood method based on JTT matrix model.** SYD_ECO45**:**B7MBS5; SYD_PYRAB**:**Q9V036; SYDC_HUMAN**:**P14868; SYDM_HUMAN**:**Q6PI48; SYD_META3**:**A6USZ9; SYD_METAC**:**Q8TQ68; SYD_METBF**:**Q466Y5; SYD_METBU**:**Q12XU7; SYD_HELP2**:**B6JLK1; SYD_METJA**:**Q58950; SYDC1_DICDI**:**Q75JQ1; SYDC2_DICDI**:**Q559M9; SYDC_BOVIN**:**Q3SYZ4; SYDC_CAEEL**:**Q03577; SYDC_RAT**:**P15178; SYDC_SCHPO**:**O74407; SYDC_YEAST**:**P04802; SYDM_DICDI**:**Q55C99; SYDM_RAT**:**Q3KRD0; SYDM_SCHPO**:**O94242; SYDM_YEAST**:**P15179; SYD_AQUAE**:**O67589; SYD_BACSU**:**O32038; SYD_HAEIG**:**A5UGD3; SYD_MYCGE**:**P47282; SYD_MYCS2**:**A0QWN3; SYD_MYCTU**:**Q50649; SYD_PSEAE**:**Q51422; SYD2_THETH**:**333967100; SYD1_THETH**:**333966377.

*L. major,* like other trypanosomatids encodes two AspRS enzymes (one cytosolic and a mitochondrial enzyme). Conserved Domains Database (CDD) based domain architecture suggest that the cytosolic AspRS (LmjF.30.0460) has a non-discriminating catalytic core (AsxRS which can charge Asp/Asn) while the mitochondrial copy (LmjF.21.0895) contains the canonical AspRS catalytic core (Table 1). Sequence-based phylogeny clearly suggests that *Leishmania major* encodes two Eukaryotic or Archaeal type AspRS (Non-discriminating) (Figure 3). This is further confirmed by the PFAM domain assignments. While the Bacterial AspRS contain a GAD domain inserted within the catalytic core which could probably function as an editing domain, the Archaeal/Eukaryotic AspRS lack this GAD domain and hence belong to the non-discriminating type AspRS. An Asp/Asn synthetase domain (AsxRS) can acylate either Asp or Asn in a non-discriminating manner [44,45]. Generally, a non-discriminating type AspRS is involved in the indirect pathway of Asparagine tRNA synthesis [44,45]. The indirect pathway, in addition to a non-discriminating type AspRS requires GatCAB complex which is a multiprotein complex involved in transamidation of AsptRNA^Asn^. However, *L. major* has only GatA (LmjF.16.1360) and a very distant homolog of GatC (LmjF.18.0110). This distant homolog of GatC in *L. major* is closely related to GatF of yeast [46]. Yet GatB is absent. In *P. falciparum* only GatA and GatB subunits have been reported [11]. While, the indirect pathway of tRNA(Gln) and tRNA(Asn) charging requires either an GatCAB or GatDE in bacteria and archaea respectively. It has been reported earlier that yeast requires a GatFAB for transamidation [46,47]. The GatF subunit belongs to DUF726 (Domain of unidentified function) family of PFAM. Our database searches showed that *P. falciparum* also has a homolog of GatF (PFL0295c). It is possible that *Leishmania* and *Plasmodium* have a GatFAB instead of GatCAB. However, this requires validation.

**Figure 4:**
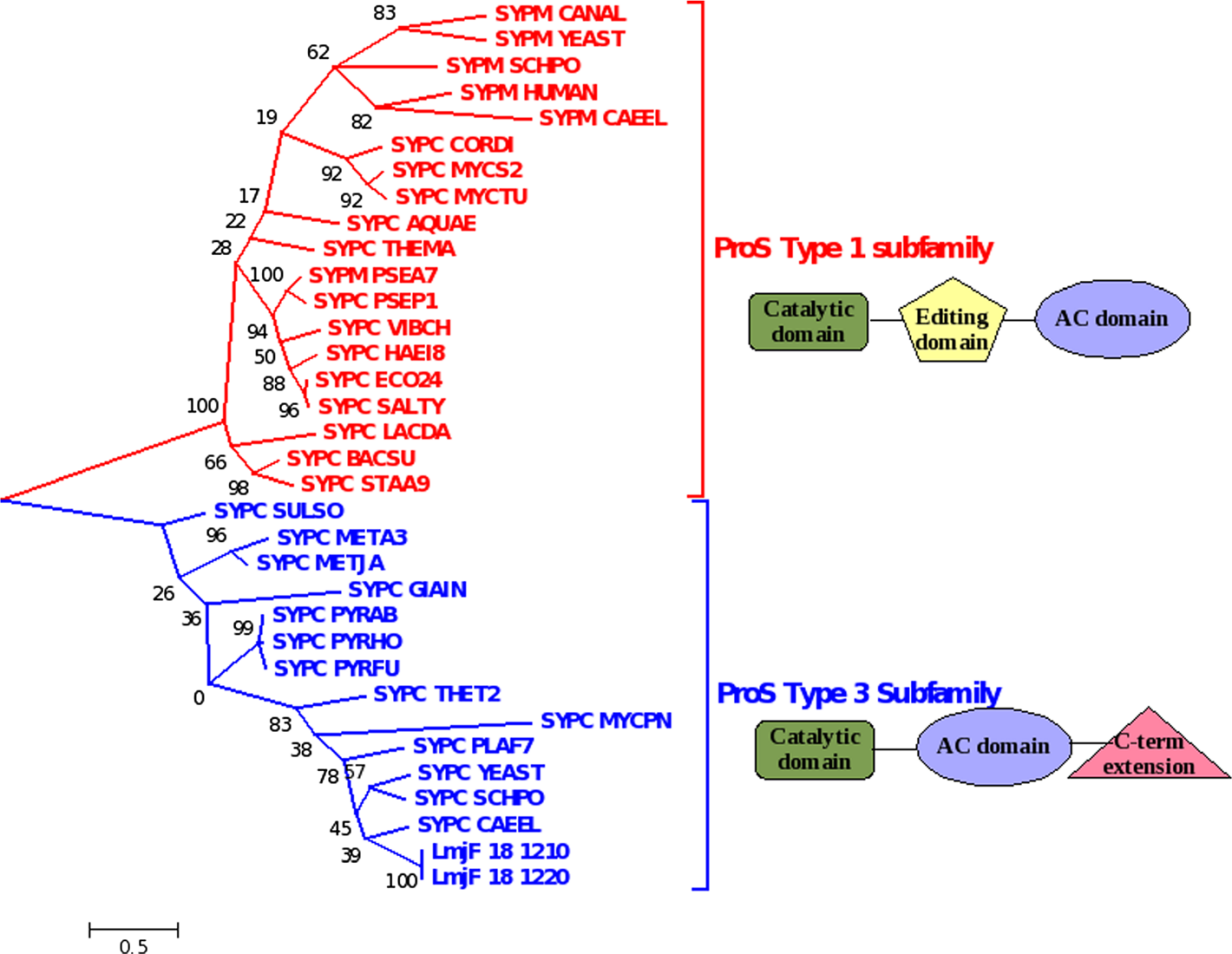
**Sequence based phylogeny of prolyl tRNA synthetases constructed using MEGA v5.0 using Maximum Likelihood method based on JTT matrix model.** SYPC_CANAL**:**P78600; SYPC_AQUAE**:**O66690; SYPC_BACSU**:**O31755; SYPC_CORDI**:**Q6NGM7; SYPC_ECO24**:**A7ZHT6; SYPC_HAEI8**:**Q4QMG5; SYPC_LACDA**:**Q1G9P3; SYPC_META3**:**A6UTK4; SYPC_MYCPN**:**P75382; SYPC_MYCS2**:**A0QVM0; SYPC_MYCTU**:**O05814; SYPC_PLAF7**:**Q8I5R7; SYPM_PSEA7**:**A6VA16; SYPC_PSEP1**:**A5VZT5; SYPC_PYRAB**:**Q9V022; SYPC_PYRFU**:**Q8U1C4; SYPC_PYRHO**:**O58734; SYPC_SALTY**:**Q7CR62; SYPC_STAA9**:**A5ISE7; SYPC_SULSO**:**Q9UWX2; SYPC_VIBCH**:**Q9KTM7; SYPM_HUMAN**:**Q7L3T8; SYPM_SCHPO**:**O74765; SYPM_YEAST**:**P39965;SYPC_THET2**:**Q72GF9; SYPC_YEAST**:**gi|500692; SYPC_METJA**:**gi|2501043; SYPC_GIAIN**:**gi|10800405; SYPC_CAEEL**:**gi|459009; SYPM_CAEEL**:**gi|3880329; SYPC_SCHPO**:**gi|3218410; SYPC_THEMA**:**gi|4981026.

There are two cytosolic ProRS enzymes both of which are annotated as bifunctional enzymes from *L. major* (LmjF.18.1210; LmjF.18.1220). Conserved Domains Database (CDD) based domain assignments suggest that they have a YbaK domain in addition to the catalytic core and anticodon binding domains (Table 1). YbaK domain has been suggested to hydrolyse misacylated tRNA^Pro^; essentially editing function [40]. Sequence based phylogeny of ProRS from all domains of life suggest that the *Lm*ProRS cluster with other cytosolic eukaryotic and archaeal ProRS all of which belong to the ProS type 3 subfamily domain architecture (according to PFAM domain assignment); while the bacteria and other eukaryotic mitochondrial enzymes have a ProS type 1 subfamily domain architecture (Figure 4). ProRS have been shown to be capable of aminoacylating both tRNA^Pro^ and tRNA^Cys^ with their respective cognate aminoacids in archaea lacking CysRS. Sequence based clustering of *Lm*ProRS with the bifunctional archaeal ProRS enzymes suggest that the bifunctional *Lm*ProRS enzymes are probably capable of charging both tRNA^Pro^ and tRNA^Cys^ with their respective aminoacids. The presence of a ProX type cis editing domain at the N-terminus of both *Leishmania* ProRS which specifically hydrolyses the misacylated tRNA^Pro^ with Alanine (Additional file 5: Figure S3) further confirms the bifunctional ability of *Lm*ProRS proteins.

### Asparagine synthetase A from *L. major –* A novel enzyme specific to Prokaryotes and Archaea

**Figure 5:**
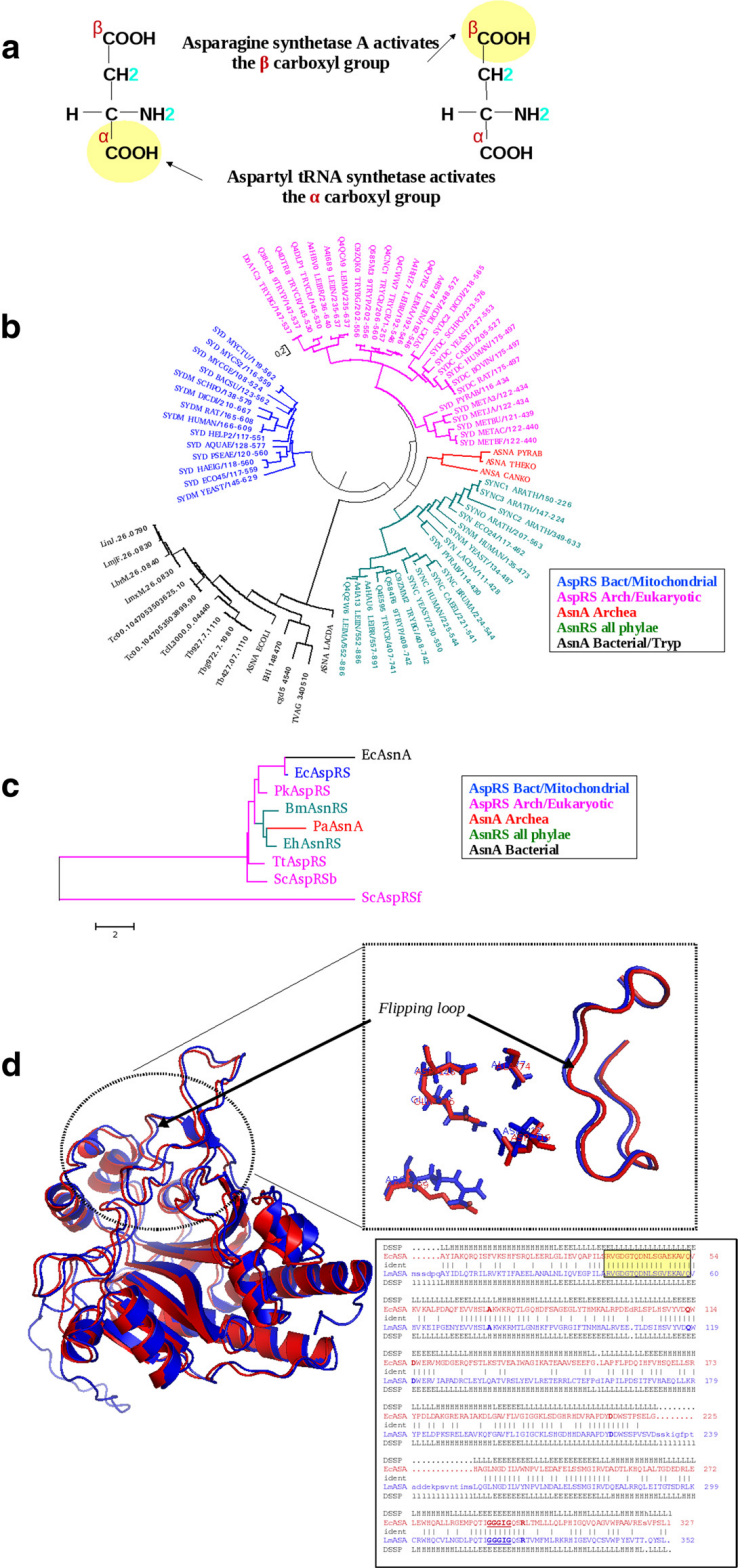
**(a) Schematic representation of the difference in the activation of L-Aspartic acid by aspartyl-tRNA synthetase (AspRS) and Asparagine synthetase A (AsnA).** (**b**) Lysyl tRNA synthetase rooted sequence based phylogeny of Asparagine synthetase A with aspartyl and asparagine tRNA synthetases. This tree is constructed using MEGA v5.0 using maximum likelihood method based on JTT matrix model. (**c**) Structure dependent sequence based phylogeny of the crystal structures of AsnA from *E. coli* (EcAsnA**:** 11AS); *P. abyssi* (PaAsnA**:** 3P8Y), AspRS from *T. kodakarensis* (TkAspRS**:** 3NEM); *T. thermophilus* (TtAspRS**:** 1EFW); *T. thermophilus* (TtAspRS2**:** 1IL2); *S. cerevisiae* (ScAspRSf**:** 1E0V) and AsnRS from *E. histolytica* (EhAsnRS**:** 3M4P); *B. malayi* (BmAsnRS**:** 2XGT). The tree is constructed using the structure based sequence alignment generated using MUSTANG structural alignment program. (**d**) A snapshot of the active site residues in *Ec*ASA (PDB**:** 11AS A chain; Red color) with *Lm*ASA structural model (blue color). Structural comparison shows the superposition of the flipping loop and active site residues from both the structures. The pairwise sequence alignment of the corresponding structural superposition with the respective color coding is also shown. The ATP binding Glycine residues are shown in BOLD, underlined fonts. The residues in the flipping loop are shown in the yellow boxes and the active site residues are shown in BOLD font.

Aminoacyl tRNA synthetase paralogs so far have been reported from prokaryotes [48]. These paralogs while retaining the aaRS catalytic domain with the characteristic motifs, are primarily involved in aminoacid biosynthesis [49,50]. Examples include AsnA, HisZ, lysylation of a specifc lysine in EF-P (Genx/PoxA) [49-54] etc. Absence of these paralogs in mammals makes them unique antibacterial drug targets. Biochemical characterization of AsnA and GenX/PoxA from *E. coli* are available [51-55]. One of the *L. major* protein (LmjF.26.0830) has a AsnRS catalytic core with all the three characteristic class II motifs conserved (Figure 4b). But, it lacks the anticodon binding domain essential for tRNA binding (Table 1). A blast sequence search against PDB database suggests close sequence similarity (~58%) with *E. coli* AsnA. Crystal structure of the *E. coli* protein indeed shows a class II tRNA synthetase core domain structure [55]. The structure based sequence comparison of yeast AspRS catalytic core with *Ec*AsnA shows conservation of structurally and catalytically important residues between the two sequences [55]. While the two substrates (mgATP and aspartic acid) of these two sequences are similar, the reactive carboxyl groups of aspartic acid are different. While in AspRS, the α-carboxyl group of aspartic acid is activated by ATP, β-carboxyl group is activated in AsnA (Figure 5a). In prokaryotes, asparagine is formed by two structurally distinct asparagine synthetases. One is the ammonia utilizing asparagine synthetase referred as AsnA and the other is the glutamine utilizing asparagine synthetase referred as AsnB. Although, AsnB can utilize glutamine or ammonia as the amide donor, glutamine is preferred over ammonia [56,57]. Recently, the crystal structure of AsnA from an archaea (*Pyrococcus abyssi*) with different substrate bound forms including AMP, asparatate, asparagine has guided in decoding the plausible mechanism of asparagine synthesis by the archaeal AsnA enzyme [58,59].

AsnA genes have been reported from prokaryotes and archaea [59,60], while AsnB genes are reported from all three domains of life. Leishmania and trypanosoma albeit being eukaryotes surprisingly possess AsnA (LmjF.26.0830) and AsnB (LmjF.29.1490). Swissprot data [18,19] suggests the presence of AsnA in almost 368 organisms all belonging to prokaryotic origin. In addition to kinetoplastids, blast searches against EupathDB database [61] suggest parasites such as *Trichomonas vaginalis (TVAG_340510; E-value: 8.9E-50); Entamoeba histolytica (EHI_148470; E-value: 1.4E-73); Cryptosporodium hominis (Chro.50501; E-value: 6.0E-26); Cryptosporodium parvum (cgd5_4540; E-value: 2.1E-52)* possess a copy of AsnA gene. *Lm*AsnA is predicted to be a mitochondrial copy (Table 1). Among the Class II synthetases, lysine, asparagine and aspartic acid are closely related in their structure and belong to the same subtype (Class 2b) [62]. However, evidences support the evolutionary link between the asparagine synthetase and aspartyl tRNA synthetases as they both recognize aspartic acid and ATP [55]. Thus, a LysRS rooted sequence based phylogeny of AsnA along with AspRS and AsnRS catalytic core from all three domains of life clearly shows that the kinetoplastid and other eukaryotic pathogen AsnA enzymes are of bacterial origin (Figure 5b) while the archaeal AsnA is derived from gene duplication events from the ancestral AspRS as previously mentioned by Blaise and workers [59]. Structure dependent sequence based phylogeny of all the available crystal structures of AspRS, AsnRS with the *Ec*AsnA and *Pa*AsnA enzymes show a similar tree branching with the *Ec*AsnA structurally closer to the bacterial AspRS (Figure 5c). The distinct branching of yeast AspRS free form clearly reflects the conformational rearrangements upon tRNA binding to the yeast AspRS [63].

To date crystal structures of three AsnA enzymes (from *E. coli, P.abyssi* and an AsnA peptide structure from *P. furious*) are available. Among the three, amino acid sequences of *L. major* AsnA and *Ec*AsnA closer to each other. Hence, a structural model of *LmA*snA was built using *Ec*AsnA (PDB:11AS) as the template using Modeller v 9.0. The model was energy minimized using Amber96 forcefield in gromacs. The quality of the energy minimized model is then verified using PROCHECK available at PDBSUM [64] webserver. Structural comparison of the *Lm*AsnA model with the *E. coli* and *P.abyssi* homologue suggests that the *Lm*AsnA shares 58% sequence identity with the bacterial homologue and 19% sequence identity with the archaeal homologue. The *L. major* model superposes with the *E. coli* and *P. abyssi* structures at 0.5Å and 2.9Å RMSD values respectively. 3-D structural comparison of the *Lm*AsnA model with the *Ec*AsnA (PDB:11AS) shows a complete conservation of catalytic residues, ATP binding Glycine rich region and identical flipping loop lengths that covers the active site at the sequence level and in 3-Dimension (Figure 5d). Based on the sequence and structural similarities between the *E. coli* and the *L. major* enzymes, D222-Q118 pair of *Lm*AsnA can be expected to anchor the beta carboxylate group of the L-aspartic acid. In yeast AspRS, these residues are substituted by a threonine and a glycine respectively and an Asp342 and Q303 which are at structurally different positions anchors the beta carboxylate group of the substrate [55]. While in *Lm*AsnA, *Ec*AsnA and yeast AspRS, an Asp-Gln pair anchors the beta carboxylate group of L-aspartic acid, in archaeal AsnA and AspRS homologues; Aspartic acid is anchored by two arginines [55]. This suggests that the altered substrate specificity and the reaction chemistry between the AspRS and AsnA have been achieved by a few residue substitutions at the active site. The basic difference in the substrate anchoring residues between the archaeal and bacterial/kinetoplastid AsnA enzymes suggests a distinct evolutionary origin between the archaeal and bacterial/kinetoplastid AsnA. These key differences between the archaeal and the kinetoplastid AsnA substrate recognition modes and the absence of AsnA in human make the kinetoplastid enzyme unique drug target for antiparasitic drug design.

## Conclusions

Aminoacyl tRNA synthetases are ubiquitous enzymes essential for cell viability. Hence, they have been one of the promising drug targets in antimicrobial infections. Due to increasing resistance to currently available anti-leishmanial drugs, aminoacyl tRNA synthetases have received attention among the kinetoplastid research community in the recent times. In this study, aminoacyl tRNA synthetases and their associated proteins from *L. major* have been explored for their novel domain architectures and sequence features. Based on the domain architecture, we identified 26 indisputable aminoacyl tRNA synthetases from *L. major*, with a predominant predicted localisation of them in the cell cytosol. Sequence based phylogeny of some specific tRNA synthetases (AspRS and ProRS) confirm their close evolutionary relationship with archaeal/eukaryotic tRNA synthetases. In addition to the appended editing domains and N- or C-terminal extensions which provide additional tRNA binding, we also identified free standing editing domains of AlaRS/ThrRS, two ProRS deacylases and two D-tyrosine deacylases (DTD). Two novel EMAP II-like sequences containing a heptapeptide motif similar to the human EMAP II-like sequences were also identified. The presence of such EMAP II-like sequences suggests the formation of a probable multisynthetase protein complex as seen in the case of human or their probable role in trans-activation of certain aminoacyl tRNA synthetases. Presence of ‘ELR’ motif in Lys, Asn, Cys and Tyr tRNA synthetases provides clues for their participation in angiogenesis likely. We also highlight the sequence analysis and 3-D structural modelling of a unique enzyme that is completely absent in human, Asparagine synthetase A from *L. major* for the first time. While the aminoacyl tRNA synthetases of *L. major* show archaeal/eukaryotic origin, Asparagine synthetase A of *L. major* shows bacterial origin. The different substrate recognition modes of the baterial and archaeal enzymes makes them unique and worth exploring.

## Methods

*Leishmania major* (Version 3.1) from TritrypDB database [65] is used here. Hidden Markov Models (HMM) [66] were generated using aminoacyl tRNA synthetase sequences and the editing domain sequences (Ybak, DTDA, AlaX) from Swissprot database Release 4.0, 2011 [67,68] for each of the 21 tRNA synthetases (20 standard tRNA synthetases + o -phosphoseryl tRNA(sec) selenium transferase) and the deacylases of ProRS (YbaK), AlaRS (AlaX) and D-tyrosine deacylase (DTDA). The distribution of aaRS sequences from the individual domains of life used for the generation of HMMs is given in the Additional file 7: Table S3. hmmbuild and hmmsearch options in the suite of HMMER 3.0 package [66] was used for generation and searches using the HMMs respectively. Multiple sequence alignment used for model generation was done using MAFFT multiple sequence alignment tool [69] which employs fast fourier transforms (FFT) for rapid identification of homologous regions. The accuracy of alignments generated by MAFFT has been proved comparable to CLUSTALW and T-coffee progressive alignment methods with the rapid reduction of CPU time [69]. BLAST Webserver at NCBI was used extensively for sequence searches against PDB database [70]. PSORT II [71] was used for subcellular localization prediction analysis. The prediction accuracy for cross validation of yeast sequences is about 57%. PSORT II does not account for multiple localization of protein sequences. PSIPRED (Protein structure prediction server) [72] is used for secondary structural prediction of the leucyl tRNA synthetase of *L. major*. PFAM database [73] from the Sanger Institute, Conserved Domains Database (CDD Server) at NCBI [74] and SUPERFAMILY database (Version 1.75) [75] were used for domain assignments. BLAST searches against PDB database was used for the assignment of deacylase (Connective peptide; CP) domains for Leu, Ile and Val tRNA synthetases.

### Phylogenetic analysis

Phylogenetic analysis of the *Lm*aaRSs was performed combining the set of sequences from the Swissprot/UniprotKB database [67]. Multiple sequence alignment (MSA) of these sequences is generated using CLUSTALW with default parameters [76]. These MSAs were used as seed sequences for phylogenetic tree generation using Jones-Taylor-Thornton (JTT) model [77]. MEGA v5 [78] was used for both analysis and visualization of the phylogenetic trees.

### Model building and validation

Comparative structural model of *L. major* Asparagine Synthetase A was built using Modeller v9 [79]. Stereochemical quality of the model was verified using PROCHECK in PDBSUM web resource at EBI [63]. Structural mapping of the active site residues was performed using Pymol [80].

## Abbreviations

aaRS: Aminoacyl tRNA synthetases; CDD: Conserved domains database; PDB: Protein data bank.

## Competing interests

The author declares that they have no competing interests.

## Authors’ contributions

VSG performed the analysis and wrote the manuscript. RMB, AS and IG analyzed the problem and reviewed the manuscript. All authors read and approved the final manuscript.

## Supplementary Material

Additional file 1 **Table S1. **Alternative splicing of aaRSs, aaRS paralogs, editing domains and other associated proteins in Promastigote stages in *Leishmania major.* Click here for file

Additional file 2 **Table S2. **Distribution of aaRSs, aaRS paralogs, editing domains and other associated proteins in *Leishmania and Trypanosoma. Organisms are Lm: L. major; Lbr: L. braziliensis; Lta: L. tarentolae; Lmx: L. mexicana; Lin: L. infantum; Tb427: T.brucei brucei427; Tb927: T. brucei brucei (TREU927); Tbg: T. brucei gambiense; TcEs: T.cruzi Esmeraldo; TcNEs: T. cruzi Non-Esmeraldo; Tcon: T. congolense; Tv: T. vivax.* Click here for file

Additional file 3 **Figure S1. **Secondary structure prediction of *Lm*LeuRS showing the first 300 residues which includes the CP1 domain. The 35 residue N-terminal insertion in the CP1 domain is highlighted in yellow. The numerical values corresponds to the confidence levels (ranging from 0–9). H refers to Helix; C refers to Coil and E refers to Sheet. Click here for file

Additional file 4 **Figure S2.** Sequence based phylogeny of tRNA_SAD domains (cis/trans) of alanyl tRNA synthetases constructed using MEGA v5.0 using Maximum Likelihood method based on JTT matrix model. Bootstrap values are indicated at the inner nodes. Click here for file

Additional file 5 **Figure S3. **Sequence based phylogeny of prolyl tRNA synthetase editing domains (Ybak/ProX) constructed using MEGA v5.0 using Maximum Likelihood method based on JTT matrix model. Bootstrap values indicated at the inner nodes indicate the similarity of tethered editing domains of *Lm*ProRS to AlatRNA^Pro^ type editing domains (ProX) and the standalone editing domains of *Lm*ProRS to CystRNA^Pro^ type (Ybak). Click here for file

Additional file 6 **Figure S4. **Sequence based phylogeny of D-tyrosine deacylases (Dtdas) from DTDA2_HUMAN (sp|Q96FN9); DTD2_MOUSE (sp|Q8BHA3); DTD2_DANRE (sp|Q68EL2); DTD1_ECOLI (1JKE); DTD2_HINF (1J7G); DTD1_AAQU (2DBO); DTD1_PFAL (3K05); DTD1_BOVIN (sp|Q2T9V8); DTD1_HUMAN (2OKV);GEK1_ARATH (sp|Q9ZPQ3); DTDA_PYRFU (sp|P58852); DTDA_PYRHO (sp|O57774); DTDA_PYRAB (sp|Q9V2R8) constructed using MEGA v5.0 using Maximum Likelihood method based on JTT matrix model. Bootstrap values are indicated at the nodes. Click here for file

Additional file 7 **Table S3. **Distribution of aaRS sequences used for generation of HMMs for all the 21 aaRS from the Swissprot database in Bacteria, Archea, Eukaryotes and Virus is given in the table. Click here for file
