# Supplementary material for: Unusual domain architecture of aminoacyl tRNA synthetases and their paralogs from Leishmania major
Source: BMC Genomics. 2012 Nov 14;13:621. doi: 10.1186/1471-2164-13-621 (PMC3532385; doi:10.1186/1471-2164-13-621)
Supplement: Additional file 3 — Figure S1. Secondary structure prediction of LmLeuRS showing the first 300 residues which includes the CP1 domain. The 35 residue N-terminal insertion in the CP1 domain is highlighted in yellow. The numerical values corresponds to the confidence levels (ranging from 0–9). H refers to Helix; C refers to Coil and E refers to Sheet. [file 1471-2164-13-621-S3.pdf]

Additional Figure 1

Secondary structure prediction of *Lm*LeuRS showing the first 300 residues which includes the CP1 domain. The 35 residue N-terminal insertion in the CP1 domain is highlighted in yellow. The numerical values corresponds to the confidence levels (ranging from 0-9). H refers to Helix; C refers to Coil and E refers to Sheet.

```
Conf: 983012346999999999998959983159999999999954995789988875440000 2
Pred: CCCCCCCCHHHHHHHHHHHHHHCCCCCCCCCCCCCCCCCCEEEECCCCCCCCCCCCCCCCC
  AA: MSTARRDALVAIEQEKQAYWAAEKLHEFDAPAPGEARPAKYLTTFPFPYMNGKLHLGHGF
                10        20        30        40        50        60

Conf: 246989999986408970024576787790189999999998864199999963323589
Pred: HHHHHHHHHHHHHCCCCEECCCCCCCCCCHHHHHHHHHHHHHHHHCCCCCCCCCCCCCC
  AA: SLTKAEFASRFQRMMGRRSLWPFGFHVTGTPIAACAQKIAKEMQQYGNPPQFPAELLEDK
                70        80        90       100       110       120

Conf: 999887887210123322246779980488999952999233302489578997285358
Pred: CCCCCCCCCCCCCCCCCCCCCCCCCCHHHHHHHHCCCCHHHHCCCCCHHHHHHHCCHHH
  AA: PKTPVVKEPTEALGQHKSKRGKSGPAKPQWLIMRSMGIPDSEIAKFADPQYWLDYFPPIA
               130       140       150       160       170       180

Conf: 999976157754444323115784200110044201238999999999655189842 5999
Pred: HHHHHHHCCCCCCCCCEEECCCCCCCCCCCHHHHHCCHHHHHHHHHHHHHHHCCCCCCCCC
  AA: MEDLKHFGCHIDWRRAFMTTERNPYFDRFMSTARRDALVAIEQEKQAYWAAEKLHEFDAP
               190       200       210       220       230       240

Conf: 999999995499488998886554000023579999999987608970035566787790 3
Pred: CCCCCCCCCEEEECCCCCCCCCCCCCCCCCHHHHHHHHHHHHHHCCCCCCCCCCCCCCCHH
  AA: APGEARPAKYLTTFPFPYMNGKLHLGHGFSLTKAEFASRFQRMMGRRSLWPFGFHVTGTP
               250       260       270       280       290       300
```
