# Supplementary material for: Unusual domain architecture of aminoacyl tRNA synthetases and their paralogs from Leishmania major
Source: BMC Genomics. 2012 Nov 14;13:621. doi: 10.1186/1471-2164-13-621 (PMC3532385; doi:10.1186/1471-2164-13-621)

Additional Figure 2

Sequence based phylogeny of tRNA_SAD domains (cis/trans) of Alanyl tRNA synthetases constructed using MEGA v5.0 using Maximum Likelihood method based on JTT matrix model. Bootstrap values are indicated at the inner nodes.

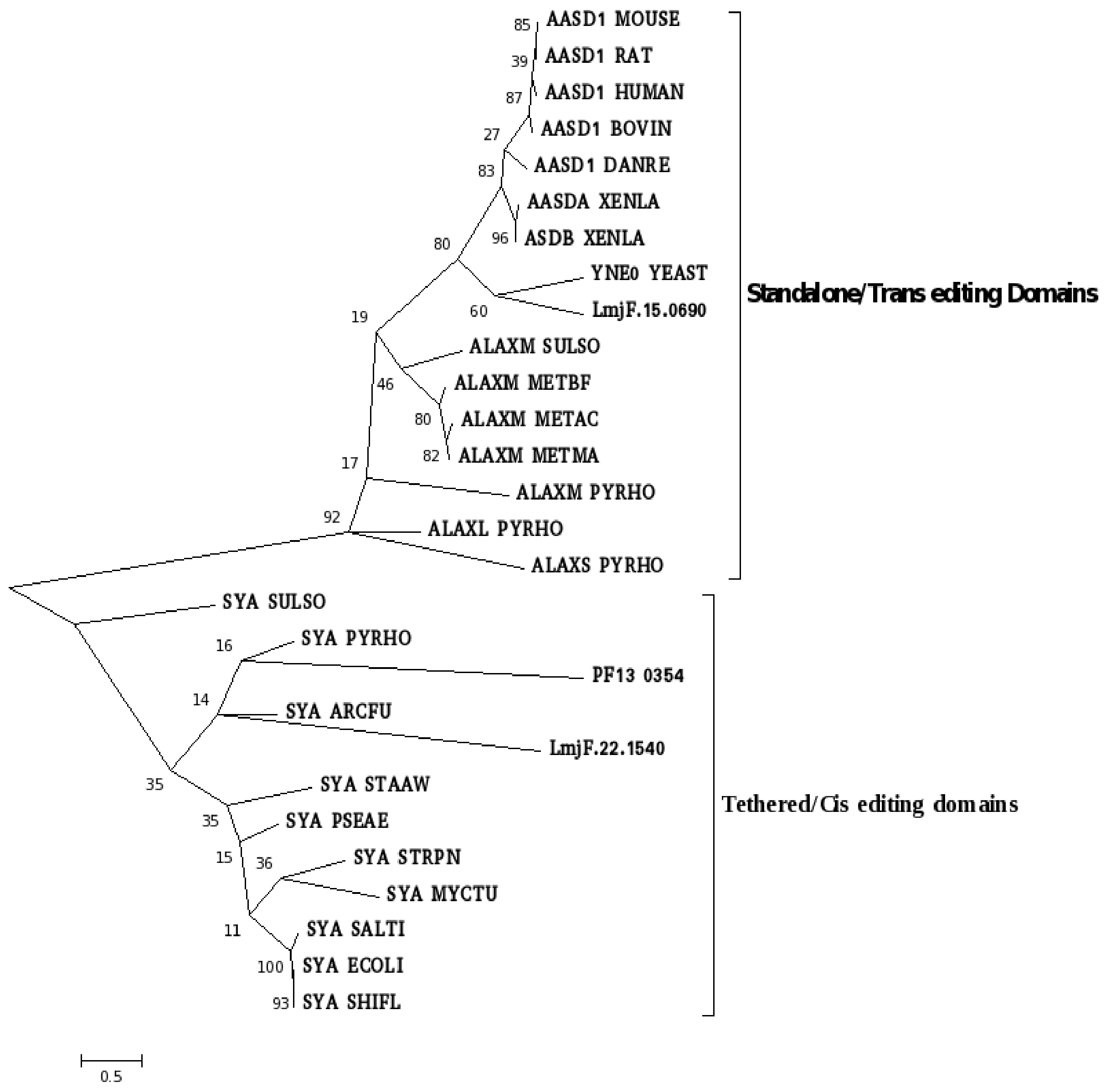

Supplement: Additional file 4 — Figure S2. Sequence based phylogeny of tRNA_SAD domains (cis/trans) of alanyl tRNA synthetases constructed using MEGA v5.0 using Maximum Likelihood method based on JTT matrix model. Bootstrap values are indicated at the inner nodes. [file 1471-2164-13-621-S4.pdf]
