# Supplementary material for: Unusual domain architecture of aminoacyl tRNA synthetases and their paralogs from Leishmania major
Source: BMC Genomics. 2012 Nov 14;13:621. doi: 10.1186/1471-2164-13-621 (PMC3532385; doi:10.1186/1471-2164-13-621)

Additional Figure 3

Sequence based phylogeny of Prolyl tRNA synthetase editing domains (Ybak/ProX) constructed using MEGA v5.0 using Maximum Likelihood method based on JTT matrix model. Bootstrap values indicated at the inner nodes indicate the similarity of tethered editing domains of *Lm*ProRS to AlatRNA^{Pro} type editing domains (ProX) and the standalone editing domains of *Lm*ProRS to CystRNA^{Pro} type (Ybak).

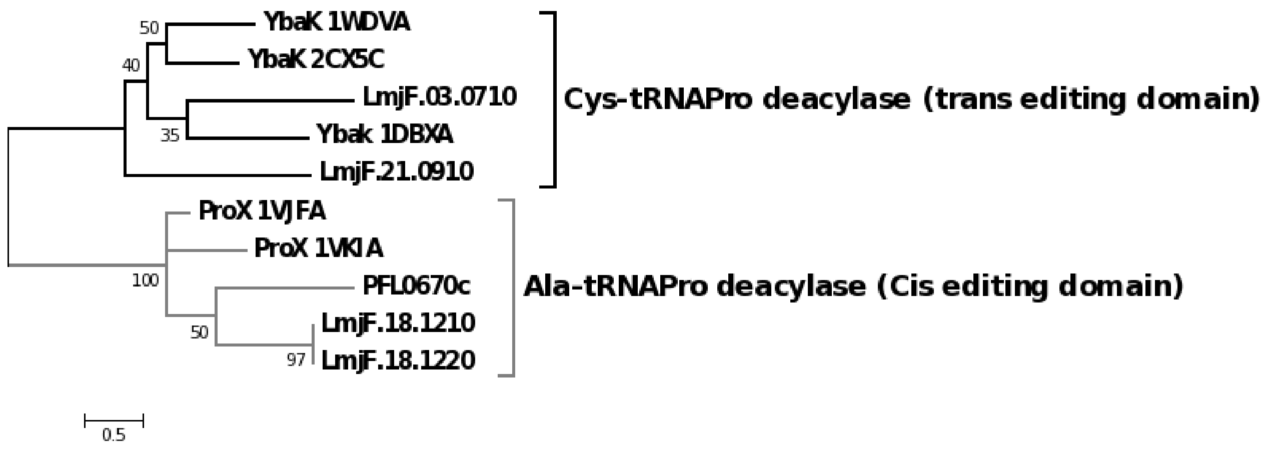

Supplement: Additional file 5 — Figure S3. Sequence based phylogeny of prolyl tRNA synthetase editing domains (Ybak/ProX) constructed using MEGA v5.0 using Maximum Likelihood method based on JTT matrix model. Bootstrap values indicated at the inner nodes indicate the similarity of tethered editing domains of LmProRS to AlatRNAPro type editing domains (ProX) and the standalone editing domains of LmProRS to CystRNAPro type (Ybak). [file 1471-2164-13-621-S5.pdf]
