# Supplementary material for: Unusual domain architecture of aminoacyl tRNA synthetases and their paralogs from Leishmania major
Source: BMC Genomics. 2012 Nov 14;13:621. doi: 10.1186/1471-2164-13-621 (PMC3532385; doi:10.1186/1471-2164-13-621)

Additional Figure 4

Sequence based phylogeny of D-tyrosine deacylases (Dtdas) from DTDA2_HUMAN (sp|Q96FN9); DTD2_MOUSE (sp|Q8BHA3); DTD2_DANRE (sp|Q68EL2); DTD1_ECOLI (1JKE); DTD2_HINF (1J7G); DTD1_AAQU (2DBO); DTD1_PFAL (3K05); DTD1_BOVIN (sp|Q2T9V8); DTD1_HUMAN (2OKV);GEK1_ARATH (sp|Q9ZPQ3); DTDA_PYRFU (sp|P58852); DTDA_PYRHO (sp|O57774); DTDA_PYRAB (sp|Q9V2R8) constructed using MEGA v5.0 using Maximum Likelihood method based on JTT matrix model. Bootstrap values are indicated at the nodes.

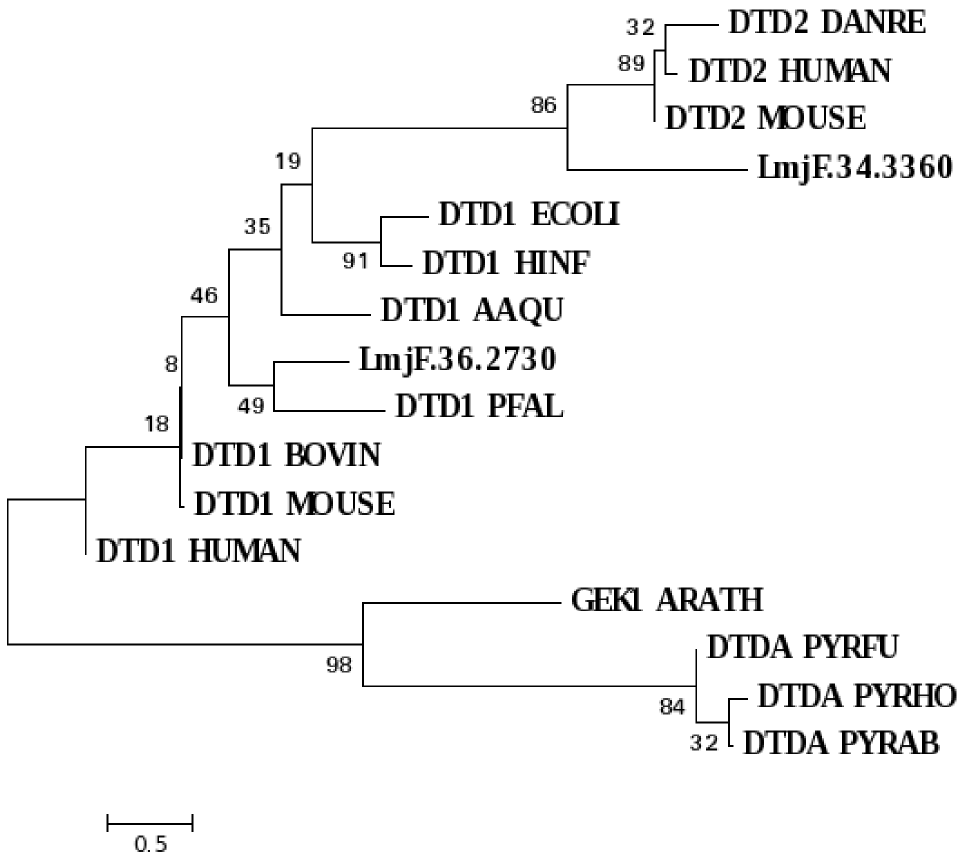

Supplement: Additional file 6 — Figure S4. Sequence based phylogeny of D-tyrosine deacylases (Dtdas) from DTDA2_HUMAN (sp|Q96FN9); DTD2_MOUSE (sp|Q8BHA3); DTD2_DANRE (sp|Q68EL2); DTD1_ECOLI (1JKE); DTD2_HINF (1J7G); DTD1_AAQU (2DBO); DTD1_PFAL (3K05); DTD1_BOVIN (sp|Q2T9V8); DTD1_HUMAN (2OKV);GEK1_ARATH (sp|Q9ZPQ3); DTDA_PYRFU (sp|P58852); DTDA_PYRHO (sp|O57774); DTDA_PYRAB (sp|Q9V2R8) constructed using MEGA v5.0 using Maximum Likelihood method based on JTT matrix model. Bootstrap values are indicated at the nodes. [file 1471-2164-13-621-S6.pdf]
